# The effect of acarbose on lipid profiles in adults: a systematic review and meta-analysis of randomized clinical trials

**DOI:** 10.1186/s40360-023-00706-6

**Published:** 2023-11-22

**Authors:** Mohsen Yousefi, Sahand Tehrani Fateh, Mahlagha Nikbaf-Shandiz, Fatemeh Gholami, Samira Rastgoo, Reza Bagher, Alireza Khadem, Farideh Shiraseb, Omid Asbaghi

**Affiliations:** 1 Faculty of Medicine, Shahid Beheshti University of Medical Sciences, Tehran, Iran; 2 School of Medicine, Tehran University of Medical Sciences, Tehran, Iran; 3 Student Research Committee, Tabriz University of Medical Sciences, Tabriz, Iran; 4 Department of Community Nutrition, School of Nutritional Sciences and Dietetics, Tehran University of Medical Sciences (TUMS), Tehran, Iran; 5 Department of Cellular and Molecular Nutrition, National Nutrition and Food Technology Research Institute, Faculty of Nutrition Science and Food Technology, Shahid Beheshti University of Medical Sciences, Tehran, Iran; 6 Student Research Committee, Shahid Beheshti University of Medical Sciences, Tehran, Iran; 7 Department of Exercise Physiology, University of Isfahan, Isfahan, Iran; 8 Department of Nutrition, Science and Research Branch, Islamic Azad University, Tehran, Iran; 9 Cancer Research Center, Shahid Beheshti University of Medical Sciences, Tehran, Iran

**Keywords:** Acarbose, Lipid profiles, Cardiovascular risk, Systematic review, meta-analysis

## Abstract

**Purpose:**

Dyslipidemia, characterized by elevated levels of triglycerides (TG), low-density lipoprotein (LDL), total cholesterol (TC), and reduced levels of high-density lipoprotein (HDL), is a major risk factor for cardiovascular diseases (CVD). Several studies have shown the potential of acarbose in improving serum lipid markers. However, there have been conflicting results on the topic in adults. Therefore, a comprehensive systematic review and meta-analysis was conducted to assess the impact of acarbose on lipid profiles.

**Methods:**

The random-effects approach was used to combine the data, and the results were provided as weighted mean difference (WMD) with 95% confidence intervals (CI).

**Results:**

Our meta-analysis included a total of 74 studies with a combined sample size of 7046 participants. The results of the analysis showed that acarbose resulted in a reduction in levels of TG (WMD = − 13.43 mg/dl, 95% CI: − 19.20, − 7.67; *P* < 0.001) and TC (WMD = − 1.93 mg/dl, 95% CI: − 3.71, − 0.15; *P* = 0.033), but did not affect other lipid markers. When conducting a nonlinear dose-response analysis, we found that acarbose was associated with an increase in levels of HDL (coefficients = 0.50, *P* = 0.012), with the highest increase observed at a dosage of 400 mg/d. Furthermore, our findings suggested a non-linear relationship between the duration of the intervention and TC (coefficients = − 18.00, *P* = 0.032), with a decline observed after 50 weeks of treatment.

**Conclusion:**

The findings of this study suggest that acarbose can reduce serum levels of TG and TC. However, no significant effects were observed on LDL or HDL levels.

## Background

Dyslipidemia, characterized by elevated triglyceride (TG), low-density lipoprotein cholesterol (LDL), total cholesterol (TC), and reduced high-density lipoprotein (HDL) levels, is a major risk factor for cardiovascular diseases (CVD) [1]. Increased TC levels, in particular, are linked to ischemic heart disease, which was reported to be responsible for 2.6 million deaths worldwide in 2012 [2]. In 2008, 39% of adults had been diagnosed with high TC levels [2]. Individuals with dyslipidemia are twice as likely to develop CVD [3]. CVD is a rising global health concern and is a leading cause of mortality [4].

Different strategies are applied to control chronic diseases, particularly dyslipidemia, including lifestyle, diet modification, and medications [5–8]. The common medications include statin, Ezetimibe, and proprotein convertase subtilisin/Kexin type 9 **(**PCSK9) inhibitors [5]. Acarbose belongs to the class of alpha-glucosidase inhibitors, which act by inhibiting the breakdown of carbohydrates in the small intestine, thus slowing down the digestion and absorption of glucose. Therefore, it effectively prevents a rapid increase in postprandial blood glucose levels in diabetic patients. Acarbose has been widely used in the management of type 2 diabetes mellitus (T2DM) and has demonstrated its efficacy in improving glycemic control in several clinical studies [9]. The research has reported the beneficial effects of acarbose on serum levels of lipids markers [10]. In one study, which involved 84 patients with T2DM, treatment with acarbose resulted in a significant increase in HDL levels and a decrease in TG levels, while its effect on TC and LDL was not significant [11]. Similarly, in another randomized controlled trial (RCT) on 82 patients with coronary artery disease, treatment with 100 mg of acarbose led to a significant reduction in TG levels, but changes in TC, HDL, and LDL were not significant compared to the control group [12].

Hanefeld et al. conducted a meta-analysis on T2DM patients and reported a significant reduction in TG levels with acarbose treatment [13]. However, Van de Laar et al. found no significant effect of acarbose on lipid markers, including TG, TC, LDL, and HDL [14]. Monami et al. conducted a systematic review and meta-analysis and reported a significant impact of acarbose in reducing TG levels while increasing HDL levels [15]. Zhang et al. conducted a meta-analysis on patients with polycystic ovary syndrome and showed that acarbose significantly reduced TG levels while increasing HDL levels [16].

The available literature on the effect of acarbose on lipid markers has yielded inconsistent resultsHowever, there is a lack of comprehensive review and meta-analysis studies that have examined this issue. Moreover, new studies have been published recently that need to be taken into account. Therefore, a new systematic review and meta-analysis is warranted to investigate the effect of acarbose on lipid markers in adults. This review study aims to analyze RCTs that have examined the effect of acarbose on lipid markers including TG, TC, LDL, and HDL across all health conditions in adults.

## Methods

### Search strategy

The Preferred Reporting Items for Systematic Reviews and Meta-Analysis (PRISMA) guideline was used in the current systematic review and meta-analysis [14]. This meta-analysis was registered at PROSPERO (CRD42022352808). RCTs without time and language limitations were sought in the databases, including PubMed, Scopus, and Web of Science from inception to April 2023. The PICO (Participant, Intervention, Comparison/Control, Outcome) stands for Participants (healthy and unhealthy adults), Intervention (acarbose intake), Comparison (placebo/Control group), Outcome (changes in TG, TC, LDL, HDL) framework was used to search components related to the effect of acarbose on lipid markers. The reference list found at the end of the articles was searched to avoid missing any articles. A combination of MeSH terms, non-Mesh terms, and keywords was used. The keywords include (Acarbose) AND (Intervention OR “intervention study” OR “intervention studies” OR “controlled trial” OR randomized OR random OR randomly OR placebo OR “clinical trial” OR RCT OR blinded OR “double blind” OR “double blinded” OR trial OR “clinical trial” OR trials OR “pragmatic clinical trial” OR “cross-over studies” OR “cross-over” OR “cross-over study” OR “parallel study” OR “parallel trial” were manually searched. The identified articles were transported into the Endnote and duplicated studies were excluded.

### Study selection

The inclusion criteria were considered 1) examining the effect of acarbose on TG, TC, LDL, and HDL; 2), randomized controlled trials (parallel or cross-over design, double or single-blind), the availability of comparison (no intervention/other drugs/placebo) group; 3) adults ≥18 years old; 4) acarbose intake for over one week; 5) availability of mean or mean differences with standard deviation (SD), standard error (SE) or 95% confidence intervals (95% CI). The exclusion criteria were 1) examining the effect of other intake/medications other than acarbose on TG, TC, LDL, and HDL; 2) other study design apart from clinical trials, including animal or in vitro/in vivo studies; 3) the study population including children/adolescent (< 18 years old). The acarbose is a drug that is taken orally. Its consumption amount has been different in different studies, which according to the studies is minimum 50)mg/d(and maximum 400)mg/d(. The acarbose dosages were converted to g/d if mg/d or other units were recorded. All the abstracts in conferences, interviews, and books were excluded. If the relevant data was missed in the articles, the corresponding authors were contacted through emails. If no response was delivered, the article was not included. All articles were screened according to their titles/abstracts and full-text. Two independent reviewers extracted the relevant results. The validity of the qualifying studies to demonstrate the eligibility of studies was examined. Any dispute was resolved by discussion.

### Data extraction

After confirming the eligibility of articles by two independent reviewers, the following information was extracted: The first author’s name, country, and publication year, study design, the sample size included in the final analysis of the intervention and placebo groups, randomization, blinding, mean age, mean body mass index (BMI), sex, intervention duration, dosage and the type of intake and placebo, the participants status, confounders adjustment, adverse effects, mean or mean difference and SD or SE for the outcomes including TG, TC, LDL, HDL at the initial and end of the intervention in the intervention and placebo groups. The mean and SD for TG, TC, LDL, and HDL at the beginning and end of each intervention (for parallel and cross-over trials) were recorded. The information from both crude and adjusted models was extracted.

If there was no access to mean and SD, the mean difference was calculated by subtracting the mean value before the intervention from the mean value after the intervention. If the trial was conducted more than twice, only information from the initial and end of the study was recorded. If multiple interventions were performed, the intervention group with acarbose and the associated placebo group were included. A separate study was considered if clinical trials with two or more eligible arms were included.

### Quality assessment

Two independent reviewers examined the quality of studies using the Cochrane Collaboration tool [17]. Any conflict was rectified by discussion. Seven components were considered to determine the quality of studies: randomization sequence generation, allocation concealment, participant and researcher blindness, outcome assessor blinding, inadequate findings, and selective reporting. Studies were categorized into three groups based on the risk of bias: a high risk of bias, a low risk of bias, and an uncertain risk of bias (Table 2).

### Statistical analysis

Data analysis was performed using Stata version 11, and a *P*-value < 0.05 was considered statistically significant. The results were reported as mean differences and a 95% CI. The mean differences in TG, TC, LDL, and HDL between acarbose and placebo groups were calculated at the initial and end of the studies. If SE was available, the Hozo et al. method was applied to transform standard errors (SEs), 95% CIs, and interquartile ranges (IQRs) into SDs [18]. The SD was measured using the following formula: SD = square root [(SD at baseline)^2^ + (SD at the end of study)^2^ − (2 r × SD at baseline ×SD at the end of study)] [19]. Also, in the studies where SE was reported, the formula SD=SEM× square root (n = the number of sample size in each group) was used to measure SD. A correlation coefficient of 0.8 was considered for r [17]. A random effects model with DerSimonian and Laird method was applied to pool the findings [20]. If the values were presented in graphic forms, plot digitizers software was used to extract the information. Cochran’s Q test and the I square (I^2^) were used to assess heterogeneity [21]. If I^2^ > 40%, the heterogeneity was considered high [22]. A sensitivity analysis was conducted to evaluate each study’s effect on the pooled effect estimate. If heterogeneity was identified, subgroup analysis was conducted to determine the heterogeneity sources. Other subgroup analyses were performed according to baseline TG (< 150, ≥150), baseline TC (< 200, ≥200), LDL (< 100, ≥100) and HDL (< 40, ≥40), trial duration (< 24, ≥24), acarbose dose (< 200, ≥200) health status (diabetic, non-diabetic), and baseline BMI [overweight (25–29.9 kg/m^2^) and obese (≥30 kg/m^2^)]. To identify any publication bias, the funnel plot, Begg’s rank correlation, and Egger’s regression tests were used [23, 24]. If any publication bias was identified, Trim and fill methods were used to correct the pooled estimates [25]. The meta-regression analysis was performed to examine the effects of acarbose dosage and duration on TG, TC, LDL, and HDL. Non-linear regression analysis was used to analyze the dose-response between acarbose intake and TG, TC, LDL, and HDL.

### Certainty assessment

The GRADE (Grading of Recommendations Assessment, Development, and Evaluation) approach was used to evaluate the overall certainty of evidence over the studies [26].

## Results

### The flow of study selection

**Fig. 1:**
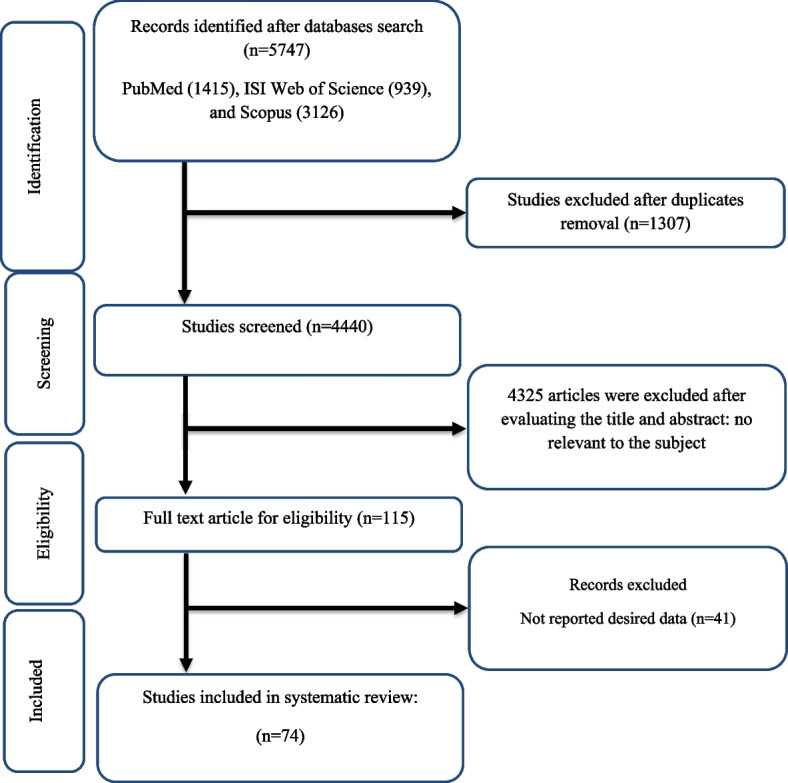
Flow chart of study selection for inclusion trials in the systematic review

The initial electronic search of the literature yielded 5747 potentially relevant citations. After duplicate removal and title/abstract screening, 115 full-text articles were retrieved for detailed assessment. Of these studies, 41 articles lacked usable data (Fig. 1). In the end, 74 studies [11, 27–99] were included in the meta-analysis. The present systematic review included 74 RCTs with a total of 7046 participants (intervention group, *n* = 3530; control group, *n* = 3516). The meta-analysis was carried out on 71, 64, 53, and 64 effect sizes for TG [11, 27, 29–32, 34–46, 48–99], TC [11, 27, 29–32, 34–36, 38–41, 43–45, 47–55, 57–65, 67–75, 77–88, 90–98], LDL [11, 33, 39–41, 43, 44, 47–49, 51, 52, 54–57, 61–65, 67–70, 72–99], and HDL [11, 27, 31, 33–35, 37–45, 47–65, 67–70, 72–91, 93–98], respectively. Except for one [95], all research was done in English.

### Study characteristics

**Fig. 2:**
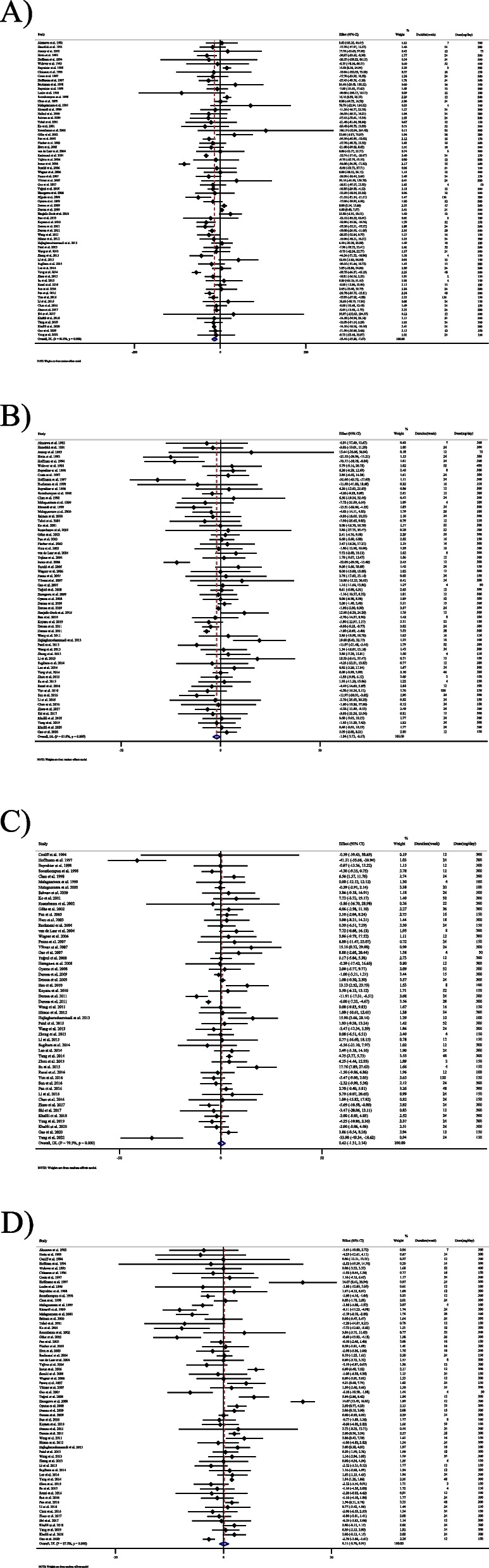
Forest plot detailing weighted mean difference and 95% confidence intervals (CIs) for the effect of acarbose on **A**) TG (mg/dl); **B**) TC (mg/dl); **C**) LDL (mg/dl) and **D**) HDL (mg/dl). Horizontal lines represent 95% CIs. Diamonds represent pooled estimates from random-effects analysis, WMD: weighted mean difference; CI, confidence interval, TG, triglyceride; TC, total cholesterol; LDL, low-density lipoprotein; HDL, high density lipoprotein

The specified characteristics of the selected studies and their study populations are summarized in Table 1. These trials were published between 1982 [27] and 2022 [99]. In total, 3530 participants were in the intervention group and 3516 participants were in the control group. Studies that were examined in this meta-analysis mostly looked at how acarbose affected lipid profiles in patients with T2DM [11, 27–36, 38–41, 43, 45, 46, 48–51, 53, 54, 58, 61, 63, 64, 69, 70, 72–75, 78, 81–87, 93, 94, 96, 97], impaired glucose tolerance [37, 55, 59, 66], obese hypertensive subjects with normal glucose tolerance [56], hypertensive T2DM patients [52], acute coronary syndrome with T2DM patients [76], nonalcoholic fatty liver disease patients [77], newly diagnosed T2DM patients [57, 65, 79, 80, 89, 91, 92], metabolic syndrome [95], obesity or overweight [42, 98], polycystic ovary syndrome [62, 67, 71, 90, 99], T2DM patients with hypercholesterolemia [60], and hypertriglyceridemia [44, 47]. These studies were carried out in Iran [77, 90, 94, 95, 98], Turkey [36, 40, 49, 64, 67], Italy [44, 45, 47, 68, 69, 73, 74], Germany [30, 34, 39, 42, 51, 53, 71], Japan [11, 31, 50, 58–60, 65, 72, 76, 83], China [27, 41, 48, 55, 63, 70, 78, 80, 81, 84–86, 88, 89, 91–93, 97, 99], Taiwan [54, 75, 87], Indiana [79], Netherlands [57, 66], Brazil [52, 62], Sweden [61], Israel [56], France [46], Thailand [43], UK [29], Spain [38], Canada [35, 37], USA [33], Australia [32], New Zealand [28], and Korea [82, 96]. Except for six studies [40, 62, 67, 71, 90, 99] that were conducted only on women and one research that was conducted exclusively on males [42], all investigations were conducted on both sexes. The intervention group in these studies consisted of 6 [32, 42, 50] to 382 [89] whose mean ages and baseline BMIs ranged from 19.31 [94] to 67.9 [60] years old and 23.4 [11, 65] to 37.26 [40] kg/m^2^, respectively. Seven studies used a cross-over design [28, 32, 36, 43, 44, 63, 94], while the others had a parallel design [11, 27, 29–31, 33–35, 37–42, 45–62, 64–93, 95–99]. The daily dosage of acarbose ranged from 50 mg [63] to 400 mg [35]. The included clinical trials’ interventions ranged in length from 2 [86] to 156 [66] weeks. Some studies used glucomannan [27], metformin [36, 58, 71, 84, 89–91, 94, 97], gliclazide [49, 80], insulin [48], pioglitazone [51, 68, 87], colestimide [60], tolbutamide [57], repaglinide [69], nateglinide [63, 78, 81, 86], glibenclamide [75], ezetimibe [77], voglibose [82], mulberry twig (Ramulus Mori, Sangzhi) alkaloid tablet [88] and placebo [28–39, 41–43, 45–47, 52–56, 59, 62, 73, 95] for control groups, and other studies used nothing. The TG, TC, LDL, and HDL forest plots showed the weighted mean difference (WMD) and 95% CI in Fig. 2A, B, C, and D respectively.

**Table 1:** Characteristics of included studies in the meta-analysis

Studies	Country	Study Design	Participant	Sample size and Sex	Sample size		Trial Duration (Week)	Means Age		Means BMI		Intervention		Adverse events
					IG	CG		IG	CG	IG	CG	Acarbose (mg/d)	Control group	
Akazawa et al. 1982 [27]	China	Parallel, R, PC	Type 2 diabetes mellitus	M/F; 24	10	14	7	20–79	20–79	NR	NR	300	Glucomannan	Intestinal side effects, such as flatulence, diarrhea and eruction (*n* = 12)
Scott et al. 1984 [28]	New Zealand	Crossover, R, PC	Non-insulin-dependent diabetes mellitus	M/F: 18	18	18	4	55.5 ± 7.1	55.5 ± 7.1	NR	NR	300	Placebo	Gastrointestinal side effects, headache
Hanefeld et al. 1991 [30]	Germany	Parallel, R, PC, DB	Non-insulin-dependent diabetes mellitus	M/F: 94	47	47	24	60 ± 16.5	59 ± 16.5	27.4 ± 7.85	27.7 ± 8.5	300	Placebo	Diarrhea and Flatulence
Jenney et al. 1993 [32]	Australia	Crossover, R, PC, DB	Non-insulin-dependent diabetes mellitus	M/F: 6	6	6	12	60.3 ± 2.5	60.3 ± 2.5	NR	NR	75	Placebo	No side effects
Hotta et al. 1993 [31]	Japan	Parallel, R, PC, DB	Non-insulin-dependent diabetes mellitus	M/F: 37	19	18	24	49.8 ± 17.5	47.9 ± 18	23.5 ± 4.15	22.9 ± 4.4	300	Placebo	Gastrointestinal symptoms
Coniff et al. 1994 [33]	USA	Parallel, R, PC, DB	Non-insulin-dependent diabetes mellitus	M/F: 189	91	98	12	56 ± 9.5	55.8 ± 10	32 ± 16.75	31.5 ± 12.3	300	Placebo	Gastrointestinal symptoms (cramp ing, abdominal pain, flatulence, and soft stools or diarrhea)
Hoffman et al. 1994 [34]	Germany	Parallel, R, PC, DB	Non-insulin-dependent diabetes mellitus	M/F: 58	28	30	24	58.8 ± 6.9	56.9 ± 6.7	26.5 ± 1.6	26.8 ± 1.5	300	Placebo	Bloating(*n* = 11) and flatulence (*n* = 12)
Wolever et al. 1995 [35]	Canada	Parallel, R, PC, DB	Diabetes mellitus	M/F: 85	41	44	52	54.4 ± 11.5	57.6 ± 9.7	31.9 ± 6.3	29.7 ± 4.5	400	Placebo	Flatulence
Chiasson et al. 1996 [37]	Canada	Parallel, R, PC, DB	Obese women with Impaired Glucose Tolerance	M/F: 18	8	10	16	56.1 ± 8.7	55.4 ± 8.7	32.2 ± 6.9	29.3 ± 2.7	150	Placebo	No statement
Bayraktar et al. 1996 [36]	Turkey	Crossover, R, PC	Non-Insulin-Dependent Diabetes Mellitus	M/F: 18	18	18	8	49	49	NR	NR	300	Metformin	gastrointestinal side effects (*n* = 12)
Costa et al. 1997 [38]	Spain	Parallel, R, PC, DB	Non-Insulin-Dependent Diabetes Mellitus	M/F: 65	36	29	24	60.2 ± 8.4	61.7 ± 9	28.7 ± 4.2	27.4 ± 3	300	Placebo	Gastrointestinal disorders
Hoffmann et al. 1997 [39]	Germany	Parallel, R, PC, DB	Non–insulin-dependent diabetes mellitus	M/F: 63	31	32	24	58.9 ± 9.4	60.2 ± 8.6	26.4 ± 2.7	26.3 ± 2.2	300	Placebo	Bloating and flatulence (*n* = 16)
Laube et al.1998 [42]	Germany	Parallel, R, PC, DB	Overweight Subject	M: 12	6	6	12	55.53 ± 8.14	50 ± 6.23	28.5 ± 2	28.1 ± 1.4	100	Placebo	No statement
Buchanan et al. 1998 [29]	UK	Parallel, R, PC, DB	Non-Insulin Dependent Diabetes	M/F: 20	9	11	16	60.1 ± 6.8	57.6 ± 8.2	NR	NR	350	Placebo	Gastrointestinal disorders (*n* = 10)
Bayraktar et al. 1998 [40]	Turkey	Parallel, R, PC	Obese women with diabetes mellitus	F: 50	25	25	12	38.12 ± 11.25	37.08 ± 9.5	34.83 ± 5.05	37.26 ± 5.9	300	Control group	Gastrointestinal side effects (*n* = 12)
Soonthornpun et al. 1998 [43]	Thailand	Crossover, R, PC, DB	Type 2 diabetes mellitus	M/F: 15	15	15	12	57.5 ± 2.6	57.5 ± 2.6	NR	NR	300	Placebo	Gastrointestinal disorders, hypoglycemia.
Chan et al. 1998 [41]	China	Parallel, R, PC, DB	Type 2 Diabetic Patients	M/F: 126	63	63	24	52.8 ± 10.2	54 ± 10	25.4 ± 3.9	25.6 ± 3.8	300	Placebo	Flatulence
Malaguarnera et al. 1999 [44]	Italy	Crossover, R, PC	Non diabetic patients with hyper- triglyceridemia	M/F: 30	30	30	4	51.1 ± 10.2	51.1 ± 10.2	27.9 ± 3.8	28 ± 3.8	100	Control group	Gastrointestinal disorders (flatulence, nausea and diarrhea, n = 6)
Riccardi et al. 1999 [45]	Italy	Parallel, R, PC, DB	Type 1 diabetes mellitus	M/F: 116	57	59	24	32.6 ± 11.78	36.3 ± 15.35	24.62 ± 3.53	24.74 ± 3.05	300	Placebo	Gastrointestinal effects
Malaguarnera et al. 2000 [47]	Italy	Parallel, R, PC	Hyper- triglyceridemia in non-diabetic patients	M/F: 30	15	15	20	60.13 ± 5.9	58.33 ± 6.7	29.23 ± 2.83	29.5 ± 3.11	100	Placebo	No statement
Halimi et al. 2000 [46]	France	Parallel, R, PC, DB	Overweight patients with Type 2 diabetes	M/F: 129	59	70	24	56 ± 9.2	55 ± 10	30.1 ± 3.3	29.7 ± 3.3	300	Placebo	Gastrointestinal disorders
Salman et al. 2000 [49]	Turkey	Parallel, R, PC	Patients with Type 2 Diabetes	M/F: 57	27	30	24	52.6 ± 9.1	56.1 ± 8.7	30.2 ± 3.8	29.2 ± 2.8	300	Gliclazide	Mild to moderate flatulence and meteorism (*n* = 8), diarrhea (n = 1), nausea and mild abdominal pain (n = 1)
Takei et al. 2001 [50]	Japan	Parallel, R, PC	Obese Type 2 patients	M/F: 15	6	9	12	56.7 ± 10.6	57.7 ± 10	28.2 ± 3.8	27.1 ± 2.4	150	Control group	Mild abdominal distention
Ko et al. 2001 [48]	China	Parallel, R, PC	Type 2 diabetes	M/F: 57	27	30	52	58.5 ± 9.9	59.1 ± 12.5	24.3 ± 3.8	24.9 ± 3.4	300	Insulin	Flatulence, diarrhea and abdominal colic (n = 5)
Rosenbaum et al. 2002 [52]	Brazil	Parallel, R, PC, DB	Hypertensive type 2 diabetic subjects	M/F: 40	20	20	22	59.8 ± 8.2	62 ± 9.7	30.3 ± 2.9	31.7 ± 3.9	300	Placebo	Hypoglycemic episodes (n = 1), flatulence (n = 6) and/or diarrhea (n = 3)
Göke et al. 2002 [51]	Germany	Parallel, R, PC	Type 2 Diabetes Mellitus	M/F: 265	136	129	26	58.8 ± 9.1	58.9 ± 9.1	30.8 ± 4.4	30.9 ± 5.3	300	Pioglitazone	Abdominal distension/ flatulence
Pan et al. 2003 [55]	China	Parallel, R, PC, DB	Subjects with impaired glucose tolerance	M/F: 252	125	127	16	53.4 ± 8.63	55.6 ± 8.31	25.6 ± 2.99	25.8 ± 3.22	150	Placebo	Gastrointestinal events (flatulence, abdomen enlarged and diarrhea),
Fischer et al. 2003 [53]	Germany	Parallel, R, PC, DB	Type 2 diabetic patients	M/F: 50	25	25	16	59.4 ± 28	58.6 ± 31.5	27.3 ± 4	27 ± 3.5	300	Placebo	No statement
Hwu et al. 2003 [54]	Taiwan	Parallel, R, PC, DB	Type 2 diabetic patients	M/F: 107	54	53	18	58.1 ± 8.4	54.7 ± 8.6	24.2 ± 3.5	23.9 ± 3.7	300	Placebo	Flatulence
Rachmani et al. 2004 [56]	Israel	Parallel, R, PC, DB	Obese hypertensive subjects with normal glucose tolerance	M/F: 56	28	28	24	52.6 ± 6.2	53.2 ± 5.7	31.2 ± 2.3	30.9 ± 2.1	150	Placebo	No statement
Van de Laar et al. 2004 [57]	Netherlands	Parallel, R, PC, DB	Newly diagnosed type 2 diabetes	M/F: 96	48	48	8	59.3 ± 7.5	57.8 ± 7.3	29.1 ± 4.6	29 ± 4.8	300	Tolbutamide	Gastrointestinal adverse events such as flatulence, diarrhea, abdominal pain or nausea (*n* = 13), headache (n = 1)
Yajima et al. 2004 [58]	Japan	Parallel, R, PC	Type 2 Diabetics	M/F: 22	11	11	12	58.7 ± 7.5	56.10 ± 7.6	25 ± 2.65	26.1 ± 2.9	300	Metformin	No statement
Inoue et al. 2006 [59]	Japan	Parallel, R, PC	Patients with impaired glucose tolerance	M/F: 40	20	20	12	NR	NR	27.5 ± 3.8	27.5 ± 4	300	Placebo	No statement
Suzuki et al. 2006 [60]	Japan	Parallel, R, PC	Patients with Type 2 Diabetes and Hypercholesterolemia	M/F: 330	16	17	24	67.9 ± 9.9	68.8 ± 12	25 ± 2.8	25.6 ± 4	150	Colestimide	No adverse event
Wagner et al. 2006 [61]	Sweden	Parallel, R, PC	Subjects With Mild Type 2 diabetes	M/F: 31	14	17	12	57 ± 3.5	54 ± 4	28.7 ± 3.3	28.7 ± 4.7	300	Control group	No statement
Penna et al. 2007 [62]	Brazil	Parallel, R, PC, DB	Obese patients with polycystic ovarian syndrome	F: 30	15	15	24	26.69 ± 1.46	25.93 ± 1.83	35.87 ± 2.6	35.04 ± 2.84	150	Placebo	Mild abdominal distention and flatulence
Yilmaz et al. 2007 [64]	Turkey	Parallel, R, PC	Type 2 diabetes	M/F: 34	15	19	24	62.6 ± 6.6	61.5 ± 12	31.3 ± 3.7	28.2 ± 5.9	300	Control group	Flatulence and bloating (*n* = 2)
Gao et al. 2007 [63]	China	Crossover, R, PC	Type 2 diabetes	M/F: 16	16	16	4	49.4 ± 6.4	49.4 ± 6.4	NR	NR	50	Nateglinide	No statement
Tuğrul et al. 2008 [67]	Turkey	Parallel, R, PC	Overweight and non-overweight patients with polycystic ovarian syndrome	F: 74	48	26	12	27.11 ± 6.04	27.11 ± 6.04	25.94 ± 5.03	26.11 ± 4.21	300	Control group	Gastrointestinal event [abdominal pain, distention, and diarrhea (n = 12)]
Nijpels et al. 2008 [66]	Netherlands	Parallel, R, PC, DB	Persons with impaired glucose tolerance	M/F: 118	60	58	156	58.5 ± 7.9	56.5 ± 7	28.4 ± 3.9	29.5 ± 3.8	300	Placebo	Abdominal pain (13.1), diarrhea (19.7%), and flatulence (44.3%)
Hasegawa et al. 2008 [65]	Japan	Parallel, R, PC	Patients with newly diagnosed type 2 diabetes	M/F: 24	13	11	12	56.3 ± 6.5	56.1 ± 6.6	23.4 ± 3.3	23.5 ± 3.3	300	Control group	No statement
Oyama et al. 2008 [11]	Japan	Parallel, R, PC	Type 2 Diabetes Mellitus	M/F: 84	41	43	52	65 ± 6	63 ± 4	23.4 ± 2.5	23.1 ± 3.2	300	Control group	No statement
Derosa et al. 2009 [69]	Italy	Parallel, R, PC, DB	Type 2 diabetic patients	M/F: 103	52	51	15	55 ± 11	53 ± 9	26.7 ± 0.7	27.2 ± 0.9	300	Repaglinide	Gastrointestinal events
Derosa et al. 2009 [68]	Italy	Parallel, R, PC, DB	Type 2 diabetic patients	M/F: 274	136	138	24	56 ± 6	56 ± 7	26.57 ± 0.7	26.85 ± 0.7	300	Pioglitazone	Gastrointestinal events
Hanjalic-Beck et al. 2010 [71]	Germany	Parallel, R, PC, DB	Patients with polycystic ovary syndrome	F: 56	29	27	12	18–43	18–43	29 ± 7.52	31.6 ± 7.77	300	Metformin	Gastrointestinal events (Abdominal disturbance, flatulence and diarrhea)
Bao et al. 2010 [70]	China	Parallel, R, PC	Type 2 diabetes	M/F: 46	24	22	8	54.7	52.6	25.28 ± 3.33	25.47 ± 2.99	100	Control group	Hypoglycemia
Koyasu et al. 2010 [72]	Japan	Parallel, R, PC	Type 2 Diabetes Mellitus	M/F: 81	42	39	52	66.1 ± 8.6	66.5 ± 8	24.9 ± 2.7	24.5 ± 3.3	150	Control group	Cardiovascular events
Derosa et al. 2011 [73]	Italy	Parallel, R, PC, DB	Type 2 diabetic patients	M/F: 188	96	92	24	56 ± 7	56 ± 7	26.6 ± 0.8	26.8 ± 0.9	300	Placebo	Nausea, gastrointestinal events
Derosa et al. 2011 [74]	Italy	Parallel, R, PC, DB	Type 2 diabetic patients	M/F: 188	96	92	28	56 ± 7	56 ± 7	26.6 ± 0.8	26.8 ± 0.9	300	Control group	Nausea, gastrointestinal events
Wang et al. 2011 [75]	Taiwan	Parallel, R, PC	Type 2 Diabetic Patients	M/F: 51	28	23	16	52.8 ± 8.2	54.7 ± 8.3	25.9 ± 3	25.3 ± 3.8	150	Glibenclamide	Abdominal distension (13.8%), back pain (3.4%) and arthralgia (6.9%)
Hirano et al. 2012 [76]	Japan	Parallel, R, PC	Acute Coronary Syndromes Patients with Type 2 Diabetes Mellitus	M/F: 44	22	22	24	65 ± 10	65 ± 11	25 ± 3.9	24.9 ± 3.8	300	Control group	No adverse events
Hajiaghamohammadi et al. 2013 [77]	Iran	Parallel, R, PC, DB	Nonalcoholic Fatty Liver Disease	M/F: 62	33	29	10	40.6 ± 10.8	40.6v2.3	30.1 ± 9.4	30.5 ± 11.9	100	Ezetimibe	No serious adverse event
Patel et al. 2013 [79]	Indiana	Parallel, R, PC, DB	Participants with early diabetes characterized	M/F: 162	81	81	52	53.6 ± 11.1	53.6 ± 11.7	35.2 ± 7.3	35.3 ± 7.1	300	Placebo	No statement
Wang et al. 2013 [80]	China	Parallel, R, PC	Patients with Newly Diagnosed Type 2 Diabetes Mellitus	M/F: 57	27	30	24	54.7 ± 8.9	55.89 ± 10.5	NR	NR	300	Gliclazide	No statement
Zheng et al. 2013 [81]	China	Parallel, R, PC	Type 2 diabetic patients	M/F: 40	20	20	4	50.3 ± 10.3	49.8 ± 9.1	25.1 ± 3	24.7 ± 3.2	150	Nateglinide	No statement
Li et al. 2013 [78]	China	Parallel, R, PC	Type 2 diabetic patients	M/F: 39	20	19	12	58.6 ± 11.1	54.6 ± 8.6	25.9 ± 2.6	26.7 ± 2.9	150	Nateglinide	No statement
Sugihara et al. 2014 [83]	Japan	Parallel, R, PC	Patients with obese type 2 diabetes	M/F: 44	22	22	12	61.8 ± 13.7	66.6 ± 13	28.6 ± 2.7	28.7 ± 3.1	300	Control group	Abdominal symptoms
Lee et al. 2014 [82]	Korea	Parallel, R, PC	Type 2 diabetes	M/F: 121	59	62	24	58.36 ± 8.59	58.73 ± 10.09	24.7 ± 3.29	24.99 ± 3.09	300	Voglibose	Gastrointestinal events and hypoglycemia
Yang et al. 2014 [84]	China	Parallel, R, PC	Type 2 diabetes	M/F: 711	361	350	48	50.6 ± 9.2	50.2 ± 9.3	25.5 ± 2.7	25.7 ± 2.6	300	Metformin	Six serious adverse events in the acarbose group (Gastrointestinal disorders)
Zhou et al. 2015 [86]	China	Parallel, R, PC	Patients with type 2 diabetes mellitus	M/F: 103	52	51	2	53.8 ± 9.3	53.9 ± 10.2	24.88 ± 2.69	25.15 ± 2.92	150	Nateglinide	No statement
Su et al. 2015 [85]	China	Parallel, R, PC	Patients with type 2 diabetes mellitus1	M/F: 95	59	36	4	55.7 ± 11	56.5 ± 10.2	27.21 ± 4.25	26.73 ± 3.11	150	Control group	No statement
Rezai et al. 2016 [90]	Iran	Parallel, R, PC, DB	Infertile Women with Polycystic Ovary Syndrome	F: 60	30	30	12	26.3 ± 4.6	26.3 ± 4.7	26.9 ± 1.8	27.3 ± 2.4	100	Metformin	Distension, Vomiting, Diarrhea, Anorexia
Yun et al. 2016 [92]	China	Parallel, R, PC	Patients with Newly Diagnosed Impaired Glucose Tolerance	M/F: 135	67	68	120	62.24 ± 5.16	61.62 ± 4.58	26.05 ± 3.24	25.82 ± 2.45	150	Control group	Severe abdominal distension and diarrhea (n = 6)
Sun et al. 2016 [91]	China	Parallel, R, PC	Newly diagnosed type 2 diabetic patients with overweight and/or obese	M/F: 108	54	54	24	53 ± 8	52 ± 6	27.07 ± 1.97	27.02 ± 1.85	300	Metformin	Mild hypoglycemic Symptoms (n = 3), Abdominal distension (n = 10)
Pan et al. 2016 [89]	China	Parallel, R, PC	Patients newly diagnosed with type 2 diabetes mellitus	M/F: 762	382	380	48	50.59 ± 9.19	50.44 ± 9.34	25.6 ± 2.57	25.67 ± 2.58	300	Metformin	No statement
Li et al. 2016 [88]	China	Parallel, R, PC, DB	Type 2 Diabetes Mellitus	M/F: 38	15	23	24	57 ± 6.7	56 ± 9.71	25.47 ± 2.61	25.67 ± 2.74	150	SZ-A	Gastrointestinal disorders
Chen et al. 2016 [87]	Taiwan	Parallel, R, PC	Type 2 diabetic patients	M/F: 60	30	30	24	67.2 ± 7.6	66.3 ± 8.8	30.1 ± 18.4	26 ± 3.4	150	Pioglitazone	Mild to moderate gastrointestinal symptoms
Ziaee et al. 2017 [94]	Iran	Crossover, R, PC	Patients with Type-1 diabetes mellitus	M/F: 40	40	40	24	19.31 ± 1.25	19.31 ± 1.25	23.96 ± 1.7	23.21 ± 1.4	300	Metformin	No statement
Shi et al. 2017 [93]	China	Parallel, R, PC	Patients with obesity and type-2 diabetes	M/F: 36	18	18	12	38.7 ± 10.3	44.4 ± 11.1	31.13 ± 2.54	31.48 ± 3.09	300	Control group	No statement
Khalili et al. 2018 [95]	Iran	Parallel, R, PC	Patients with Metabolic Syndrome	M/F: 74	32	42	24	41.2 ± 7.7	38.9 ± 9.1	NR	NR	300	Placebo	Gastrointestinal events
Yang et al. 2019 [96]	Korea	Parallel, R, PC, DB	Patients with Type 2 Diabetes Mellitus	M/F: 131	66	65	24	60.89 ± 8.9	56.55 ± 10.6	25.05 ± 4	25.39 ± 3.6	300	Control group	Spinal osteoarthritis
Khalili et al. 2020 [98]	Iran	Parallel, R, PC, DB	Obesity and Overweight	M/F: 74	32	42	24	41.25 ± 7.71	38.92 ± 9.05	38.11 ± 5.02	36.12 ± 4.66	300	Control group	Abdominal bloating, diarrhea and stool softening
Gao et al. 2020 [97]	China	Parallel, R, PC	Type 2 diabetes mellitus	M/F: 124	62	62	12	63 ± 5.25	60 ± 5.5	25.6 ± 2.6	26.42 ± 2.76	150	Metformin	Gastrointestinal events (n = 1), hypoglycemia (n = 6)
Yang et al. 2022 [99]	China	Parallel, R, PC	Patients with primary polycystic ovarian syndrome	F:92	46	46	24	26.5 ± 5.5	26.5 ± 4.5	NR	NR	150	Control group	No statement

*Abbreviations*: *IG* intervention group, *CG* control group, *DB* double-blinded, *SB* single-blinded, *PC* placebo-controlled, *CO* controlled, *RA* randomized, *NR* not reported, *F* Female, *M* Male, *NR* not reported

Age: mean age of participants; BMI: mean of body mass index

### Adverse events

Gastrointestinal symptoms were the side effects of acarbose that were most frequently reported in the studies [27–31, 33–36, 38–41, 43–46, 48–52, 54, 55, 57, 62, 64, 66–69, 71, 73–75, 82–84, 87, 88, 90–92, 95, 97, 98]. Other side effects included headache [28, 57], hypoglycemia episodes [43, 52, 70, 82, 91, 97], cardiovascular events [72], back pain [75], arthralgia [75], anorexia [90], and spinal osteoarthritis [96].

### Qualitative data assessment

Fifty-eight trials [11, 27–38, 40–42, 44–51, 53–55, 58–61, 63–65, 67, 70, 72, 75, 76, 78–89, 91–95, 97–99] were evaluated as having bad quality since more than two domains had a high risk of bias and their general risk of bias was high. 15 trials [39, 43, 52, 56, 57, 62, 66, 68, 69, 71, 73, 74, 77, 90, 96] were classified as having medium quality and had a moderate general risk of bias, and one study [98] had good quality with a low general risk of bias in terms of their quality based on the Cochrane collaboration’s tool (Table 2).

**Table 2:** Quality assessment (A summary of the risk of bias according to Cochrane  criteria)

Study	Random sequence generation	Allocation concealment	Selective reporting	Other sources of bias	Blinding (participants and personnel)	Blinding (outcome assessment)	Incomplete outcome data	General risk of bias	Quality
Akazawa et al. 1982 [27]	U	H	H	H	H	H	L	H	Bad
Scott et al. 1984 [28]	L	H	H	H	H	H	L	H	Bad
Hanefeld et al. 1991 [30]	L	H	H	H	L	U	L	H	Bad
Jenney et al. 1993 [32]	L	H	H	H	L	U	L	H	Bad
Hotta et al. 1993 [31]	L	H	H	H	L	U	L	H	Bad
Coniff et al. 1994 [33]	L	H	H	H	L	U	H	H	Bad
Hoffman et al. 1994 [34]	L	H	H	H	L	U	H	H	Bad
Wolever et al. 1995 [35]	L	H	H	H	L	U	L	H	Bad
Chiasson et al. 1996 [37]	L	H	H	H	L	U	L	H	Bad
Bayraktar et al. 1996 [36]	U	H	H	H	H	H	L	H	Bad
Costa et al. 1997 [38]	L	H	H	H	L	U	L	H	Bad
Hoffmann et al. 1997 [39]	L	H	L	H	L	U	L	M	Fair
Laube et al.1998 [42]	U	H	H	H	L	U	L	H	Bad
Buchanan et al. 1998 [29]	U	H	H	H	H	H	L	H	Bad
Bayraktar et al. 1998 [40]	U	H	L	H	H	H	L	H	Bad
Soonthornpun et al. 1998 [43]	L	H	L	H	L	U	L	M	Fair
Chan et al. 1998 [41]	L	H	L	H	L	U	H	H	Bad
Malaguarnera et al. 1999 [44]	U	H	L	H	H	H	L	H	Bad
Riccardi et al. 1999 [45]	L	H	H	H	L	U	L	H	Bad
Malaguarnera et al. 2000 [47]	L	H	H	H	L	U	L	H	Bad
Halimi et al. 2000 [46]	L	H	H	H	L	U	L	H	Bad
Salman et al. 2000 [49]	L	H	L	H	H	H	L	H	Bad
Takei et al. 2001 [50]	L	H	H	H	H	H	L	H	Bad
Ko et al. 2001 [48]	L	H	L	H	H	H	L	H	Bad
Rosenbaum et al. 2002 [52]	L	H	L	H	L	U	L	M	Fair
Göke et al. 2002 [51]	L	L	L	H	H	H	H	H	Bad
Pan et al. 2003 [55]	L	H	L	H	L	H	L	H	Bad
Fischer et al. 2003 [53]	L	H	H	H	L	U	L	H	Bad
Hwu et al. 2003 [54]	L	H	L	H	L	U	H	H	Bad
Rachmani et al. 2004 [56]	L	L	H	H	L	U	L	M	Fair
Van de Laar et al. 2004 [57]	L	L	L	H	L	U	L	M	Fair
Yajima et al. 2004 [58]	L	H	H	H	H	H	L	H	Bad
Inoue et al. 2006 [59]	U	H	H	H	H	H	L	H	Bad
Suzuki et al. 2006 [60]	L	H	H	H	H	H	L	H	Bad
Wagner et al. 2006 [61]	L	H	L	H	H	H	L	H	Bad
Penna et al. 2007 [62]	U	H	L	H	L	U	L	M	Fair
Yilmaz et al. 2007 [64]	L	H	L	H	H	H	L	H	Bad
Gao et al. 2007 [63]	L	H	L	H	H	H	L	H	Bad
Tuğrul et al. 2008 [67]	L	H	L	H	H	H	L	H	Bad
Nijpels et al. 2008 [66]	L	L	H	H	L	U	L	M	Fair
Hasegawa et al. 2008 [65]	L	H	L	H	H	H	L	H	Bad
Oyama et al. 2008 [11]	L	H	L	H	H	H	H	H	Bad
Derosa et al. 2009 [69]	L	H	L	H	L	U	L	M	Fair
Derosa et al. 2009 [68]	L	H	L	H	L	U	L	M	Fair
Hanjalic-Beck et al. 2010 [71]	L	L	H	H	L	U	L	M	Fair
Bao et al. 2010 [70]	L	L	L	H	H	H	L	H	Bad
Koyasu et al. 2010 [72]	L	H	L	H	H	H	L	H	Bad
Derosa et al. 2011 [73]	L	H	L	H	L	U	L	M	Fair
Derosa et al. 2011 [74]	L	H	L	H	L	U	L	M	Fair
Wang et al. 2011 [75]	L	L	L	H	H	H	L	H	Bad
Hirano et al. 2012 [76]	L	H	H	H	H	H	L	H	Bad
Hajiaghamohammadi et al. 2013 [77]	U	H	L	H	L	U	L	M	Fair
Patel et al. 2013 [79]	L	H	L	H	L	U	H	H	Bad
Wang et al. 2013 [80]	L	H	L	H	H	H	L	H	Bad
Zheng et al. 2013 [81]	L	H	L	H	H	H	L	H	Bad
Li et al. 2013 [78]	L	H	L	H	H	H	L	H	Bad
Sugihara et al. 2014 [83]	L	L	L	H	H	H	L	H	Bad
Lee et al. 2014 [82]	L	H	L	H	H	H	H	H	Bad
Yang et al. 2014 [84]	U	L	L	H	H	H	H	H	Bad
Zhou et al. 2015 [86]	L	H	L	H	H	H	L	H	Bad
Su et al. 2015 [85]	L	H	L	H	H	H	L	H	Bad
Rezai et al. 2016 [90]	L	H	L	H	L	U	L	M	Fair
Yun et al. 2016 [92]	L	L	H	H	H	H	L	H	Bad
Sun et al. 2016 [91]	L	L	L	H	H	H	L	H	Bad
Pan et al. 2016 [89]	L	H	H	H	H	H	L	H	Bad
Li et al. 2016 [88]	L	H	L	H	L	U	H	H	Bad
Chen et al. 2016 [87]	U	L	L	H	H	H	H	H	Bad
Ziaee et al. 2017 [94]	L	H	L	H	H	H	L	H	Bad
Shi et al. 2017 [93]	L	H	L	H	H	H	L	H	Bad
Khalili et al. 2018 [95]	L	L	L	H	H	H	H	H	Bad
Yang et al. 2019 [96]	L	L	L	H	L	U	H	M	Fair
Khalili et al. 2020 [98]	L	L	L	H	L	U	L	L	Good
Gao et al. 2020 [97]	L	L	L	H	H	H	H	H	Bad
Yang et al. 2022 [99]	L	H	H	H	H	H	L	H	Bad

*Abbreviations*. H, high risk of bias; L, low risk of bias; U, unclear risk of bias

The Cochrane Collaboration tool was used to assess the quality of studies

Bad > 2 high risk; Good < 2 high risk; Fair = 2 high risk

### Meta-analysis

#### Effect of acarbose on TG

Acarbose significantly affected TG (WMD = − 13.43 mg/dl, 95% CI: − 19.20, − 7.67; *P* < 0.001; I^2^ = 86.8%, P < 0.001; Fig. 2A), according to the findings of a pooled analysis of 71 studies (71 effect sizes) with 6980 participants for TG [11, 27, 29–32, 34–46, 48–99]. Between-study heterogeneity disappeared in studies with overweighted participants (I^2^ = 12.4%, *P* = 0.329) (Table 3).

**Table 3:** Subgroup analyses of acarbose on lipid profiles in adults

	NO	WMD (95%CI)	*P*-value	heterogeneity		
				P heterogeneity	I^2^	P between sub-groups
Subgroup analyses of acarbose on serum TG (mg/dl)						
Overall effect	71	−13.43 (− 19.20, −7.67)	**< 0.001**	< 0.001	86.8%	
Baseline TG (mg/dl)						
< 150	27	−8.40 (− 15.24, − 1.57)	**0.016**	< 0.001	72.6%	0.095
≥ 150	44	−17.00 (−24.44, −9.56)	**< 0.001**	< 0.001	84.8%	
Trial duration (week)						
<24	34	−7.96 (−18.94, 3.01)	0.155	< 0.001	86.5%	0.150
≥ 24	37	− 17.43 (− 24.21, − 10.65)	**< 0.001**	< 0.001	86.2%	
Intervention dose (mg/day)						
<300	24	−15.57 (−23.60, −7.53)	**< 0.001**	< 0.001	61.5%	0.635
≥ 300	47	−12.97 (−20.05, − 5.88)	**< 0.001**	< 0.001	88.5%	
Baselin BMI (kg/m^2^)						
Overweight (25–29.9)	10	−14.31 (−21.14, −7.48)	**< 0.001**	0.329	12.4%	0.849
Obese (> 30)	52	−15.25 (−22.19, −8.31)	**< 0.001**	< 0.001	88.5%	
Health status						
Diabetic	55	−11.04 (−17.11, −4.96)	**< 0.001**	< 0.001	78.8%	0.137
Non diabetic	16	−21.03 (−32.71, −9.35)	**< 0.001**	< 0.001	90.6%	
Age (year)						
50>	16	−6.48 (−14.13, 1.17)	0.097	< 0.001	69.3%	0.172
50<	54	−13.66 (−20.57, −6.75)	**< 0.001**	< 0.001	85.2%	
Sex						
Both	64	− 13.98 (−20.15, −7.80)	**< 0.001**	< 0.001	87.8%	0.177
Female	6	−6.19 (−18.85, 6.47)	0.338	0.072	50.6%	
Male	1	−90.00 (−190.16, 10.16)	0.078	–	–	
Subgroup analyses of acarbose on serum TC (mg/dl)						
Overall effect	64	−1.93 (−3.71, −0.15)	**0.033**	< 0.001	67.0%	
Baseline TC (mg/dl)						
< 200	30	−2.49 (−4.87, − 0.10)	**0.041**	< 0.001	71.8%	0.602
≥ 200	34	−1.51 (− 4.29, 1.25)	0.283	< 0.001	57.6%	
Trial duration (week)						
<24	33	0.18 (−2.58, 2.96)	0.894	< 0.001	58.6%	**0.030**
≥ 24	31	−3.84 (−6.20, −1.48)	**< 0.001**	< 0.001	70.0%	
Intervention dose (mg/day)						
<300	21	0.27 (−2.49, 3.03)	0.849	0.174	22.3%	0.077
≥ 300	43	−2.89 (−5.05, −0.73)	**0.009**	< 0.001	73.5%	
Baselin BMI (kg/m^2^)						
Overweight (25–29.9)	10	−4.36 (−8.72, −0.01)	0.050	0.016	55.8%	0.271
Obese (> 30)	46	−1.63 (−3.79, 0.52)	0.138	< 0.001	71.7%	
Health status						
Diabetic	52	−1.91 (−3.77, −0.05)	**0.044**	< 0.001	62.5%	0.858
Non diabetic	12	−1.33 (−7.36, 4.68)	0.663	< 0.001	79.8%	
Age (year)						
50>	15	−0.81 (−5.55, 3.93)	0.737	< 0.001	66.6%	0.744
50<	48	−1.66 (−3.46, 0.14)	0.071	< 0.001	58.5%	
Sex						
Both	59	−2.25 (−4.11, −0.39)	**0.018**	< 0.001	68.3%	0.134
Female	5	1.64 (−3.10, 6.39)	0.498	0.365	7.3%	
Subgroup analyses of acarbose on serum LDL (mg/dl)						
Overall effect	53	0.41 (−1.30, 2.14)	0.635	< 0.001	79.3%	
Baseline LDL (mg/dl)						
< 100	9	−3.31 (−13.33, 6.69)	0.186	0.012	59.0%	0.091
≥ 100	44	1.08 (−0.91, 3.09)	0.286	< 0.001	81.1%	
Trial duration (week)						
<24	25	1.96 (−0.01, 3.95)	0.052	0.024	39.3%	0.057
≥ 24	28	−1.13 (−3.63, 1.37)	0.375	< 0.001	87.0%	
Intervention dose (mg/day)						
<300	20	2.40 (−0.59, 5.40)	0.116	< 0.001	63.0%	0.105
≥ 300	33	−0.64 (−2.80, 1.50)	0.555	< 0.001	83.5%	
Baselin BMI (kg/m^2^)						
Overweight (25–29.9)	8	1.54 (−2.30, 5.39)	0.432	0.168	32.6%	0.719
Obese (> 30)	40	0.74 (−1.21, 2.71)	0.456	< 0.001	82.0%	
Health status						
Diabetic	41	0.84 (−1.21, 2.90)	0.420	< 0.001	81.9%	0.404
Non diabetic	12	−0.76 (−3.93, 2.40)	0.637	0.002	62.6%	
Age (year)						
50>	11	−1.33 (−6.08, 3.42)	0.583	< 0.001	68.9%	0.409
50<	42	0.82 (−1.06, 2.72)	0.392	< 0.001	81.1%	
Sex						
Both	48	0.76 (−1.01, 2.53)	0.400	< 0.001	79.7%	0.264
Female	5	−5.10 (−15.25, 5.04)	0.324	0.001	78.1%	
Subgroup analyses of acarbose on serum HDL (mg/dl)						
Overall effect	64	0.10 (−0.69, 0.91)	0.792	< 0.001	87.2%	
Baseline HDL (mg/dl)						
< 40	12	−0.45 (−2.31, 1.41)	0.636	< 0.001	71.6%	0.522
≥ 40	52	0.22 (−0.66, 1.11)	0.620	< 0.001	88.3%	
Trial duration (week)						
<24	32	−0.16 (−1.89, 1.57)	0.856	< 0.001	91.2%	0.597
≥ 24	32	0.35 (− 0.41, 1.11)	0.371	< 0.001	74.0%	
Intervention dose (mg/day)						
<300	22	−1.20 (−2.35, −0.04)	**0.042**	< 0.001	68.2%	**0.009**
≥ 300	42	0.80 (−0.17, 1.79)	0.107	< 0.001	88.3%	
Baselin BMI (kg/m^2^)						
Overweight (25–29.9)	10	−0.48 (−4.78, 3.80)	0.824	< 0.001	95.5%	0.711
Obese (> 30)	49	0.33 (−0.41, 1.09)	0.382	< 0.001	79.9%	
Health status						
Diabetic	51	−0.09 (−1.00, 0.80)	0.833	< 0.001	86.9%	0.462
Non diabetic	13	0.75 (−1.33, 2.84)	0.478	< 0.001	88.9%	
Age (year)						
50>	13	−0.19 (−2.44, 2.06)	0.869	< 0.001	82.4%	0.883
50<	50	−0.00 (− 0.88, 0.86)	0.985	< 0.001	87.1%	
Sex						
Both	59	−0.04 (−0.87, 0.78)	0.922	< 0.001	87.8%	0.041
Female	4	3.50 (0.68, 6.32)	**0.015**	0.163	41.4%	
Male	1	−3.80 (−12.64, 5.04)	0.400	–	–	

*Abbreviations*: *BMI* body mass index, *CI* confidence interval, *HDL* high density lipoprotein, *LDL* low-density lipoprotein, *TC* total cholesterol, TG triglyceride, *WMD* weighted mean differences

Subgroup analyses have done

*P* < 0.05 was considered a significance and bolded

Acarbose consumption lowered TG in all subgroups according to baseline TG < 150 mg/dl (WMD = − 8.40; 95% CI: − 15.24, − 1.57; *P* = 0.016), > 150 mg/dl (WMD = − 17.00; 95% CI: − 24.44, − 9.56; *P* < 0.001), trial duration ≥24 weeks (WMD = − 17.43; 95% CI: − 24.21, − 10.65; P < 0.001), both intervention dose < 300 mg/d (WMD = − 15.57; 95% CI: − 23.60, − 7.53; P < 0.001), ≥300 mg/d (WMD = − 12.97; 95% CI: − 20.05, − 5.88; P < 0.001), BMI categories, in overweight (WMD = − 14.31; 95% CI: − 21.14, − 7.48; *P* < 0.001), and obese individuals (WMD = − 15.25; 95% CI: − 22.19, − 8.31; P < 0.001). adults older than 50 years (WMD = − 13.66; 95% CI: − 20.57, − 6.75; P < 0.001), studies on both sexes (WMD = − 13.98; 95% CI: − 20.15, − 7.80; P < 0.001). Moreover, in both health statuses including diabetic patients (WMD = − 11.04; 95% CI: − 17.11, − 4.96; P < 0.001) and non-diabetic (WMD = − 21.03; 95% CI: − 32.71, − 9.35; P < 0.001).

#### Effect of acarbose on TC

In total, 64 effect sizes from 64 trials were considered in this analysis, representing a population of 5590 participants. After consuming acarbose, pooled effect sizes showed a substantial drop in TC (WMD = − 1.93 mg/dl, 95% CI: − 3.71, − 0.15; *P* = 0.033; I^2^ = 67%, *P* < 0.001; Fig. 2B). When trials utilized less than 300 mg of acarbose, between-study heterogeneity was eliminated (I^2^ = 22.3%, *P* = 0.174).

Acarbose significantly reduced TC in high-dose interventions (≥300 mg/d), according to subgroup analyses (WMD = − 2.89; 95% CI: − 5.05, − 0.73; *P* = 0.009), and in studies with ≥24 weeks of intervention (WMD = − 3.84; 95% CI: − 6.20, − 1.48; *P* < 0.001 (Table 3). Other subgroup analyses based on health status and baseline TC also showed that acarbose significantly reduced TC in diabetic patients (WMD = − 1.91 mg/dl, 95% CI: − 3.77, − 0.05; *P* = 0.044), individuals with baseline TC < 200 (WMD = − 2.49 mg/dl, 95% CI: − 4.87, − 0.10; *P* < 0.041) and studies on both sexes (WMD = − 2.25; 95% CI: − 4.11, − 0.39; *P* = 0.018).

#### Effect of acarbose on LDL

Fifty-three trials (*n* = 5970) measured the effect of acarbose on LDL [11, 33, 39–41, 43, 44, 47–49, 51, 52, 54–57, 61–65, 67–70, 72–99]. Overall, we observed no difference in LDL reduction between the intervention and control groups (WMD = 0.41 mg/dl, 95% CI: − 1.30, 2.14; *P* = 0.635; I^2^ = 79.3%, P < 0.001; Fig. 2C). Between-study heterogeneity was eliminated in studies with overweight participants (I^2^ = 32.6%, *P* = 0.168) (Table 3). There was not any significant relation between subgroups and LDL changes (*P* > 0.05).

#### Effect of acarbose on HDL

Changes in HDL were assessed in 64 trials (*n* = 6318) [11, 27, 31, 33–35, 37–45, 47–65, 67–70, 72–91, 93–98]. The variations in HDL when compared to controls were not significant (WMD = 0.10; 95% CI: − 0.69, 0.91; *P* = 0.792; I^2^ = 87.2%, P < 0.001; Fig. 2D). However, in subgroup analysis, acarbose resulted in decreases (WMD = − 1.20; 95% CI: − 2.35, − 0.04; *P* = 0.042) in the low-dose intervention (< 300 mg/d), and increase in females (WMD = 3.50; 95% CI: 0.68, 6.32; *P* = 0.015) (Table 3).

### Nonlinear dose-response analysis

In the non-linear dose-response analysis, there was evidence of a non-linear connection between acarbose dosage and HDL (coefficients = 0.50, *P* = 0.012; Fig. 4D), with the biggest increase in dosage being 400 mg/d acarbose. However, no evidence of a nonlinear relationship between acarbose dosage and TG (coefficients = − 5.10, *P* = 0.586; Fig. 4A), TC (coefficients = − 14.91, *P* = 0.187; Fig. 4B), or LDL (coefficients = − 3.72, *P* = 0.345; Fig. 4C) was found. There was no evidence of a non-linear association between the duration of the intervention and TG (coefficients = 24.12, *P* = 0.189; Fig. 5A), LDL (coefficients = 2.19, *P* = 0.118; Fig. 5C), and HDL (coefficients = 1.76, *P* = 0.426; Fig. 5D), according to the results of the non-linear dose-response analyses. However, there was a non-linear association between duration of intervention and TC with the highest reduction after 50 weeks (coefficients = − 18.00, *P* = 0.032; Fig. 5B).

### Meta-regression analysis

To evaluate how acarbose and the duration of the intervention changed lipid profiles, a meta-regression analysis was employed. No significant linear association between changes in TC (coefficients = − 0.30, *P* = 0.238; Fig. 6B), LDL (coefficients = − 0.19, *P* = 0.505; Fig. 6C), and HDL (coefficients = 0.13, *P* = 0.741; Fig. 6D) and duration existed. However, we found a significant linear association between TG (coefficients = − 0.28, *P* = 0.044; Fig. 6A) and duration of intervention.

We discovered a significant linear association between the intervention’s dose (g/d) (coefficients = 5.54, P = 0.032; Fig. 7D) and changes in HDL. Acarbose dose and changes in other variables did not have a significant linear association (Fig. 7 A, B and C).

### Sensitivity analysis

Findings regarding acarbose consumption and TG, LDL, and HDL remained robust in the sensitivity analysis. However, the significant effect of acarbose on TC disappeared when excluding the studies by Hotta et al. [31] (WMD = − 1.68, 95% CI: − 3.44, 0.07), Hoffman et al. [34] (WMD = − 1.72, 95% CI: − 3.49, 0.04), Hoffmann et al. [39] (WMD = − 1.62, 95% CI: − 3.36, 0.10), Riccardi et al. [45] (WMD = − 1.72, 95% CI: − 3.51, 0.06), Inoue et al. [59] (WMD = − 1.45, 95% CI: − 3.13, 0.22), Derosa et al. [68] (WMD = − 1.92, 95% CI: − 3.89, 0.04), Derosa et al. [74] (WMD = − 1.75, 95% CI: − 3.54, 0.04), Patel et al. [79] (WMD = − 1.75, 95% CI: − 3.54, 0.03), and Sun et al. [91] (WMD = − 1.74, 95% CI: − 3.53, 0.04).

### GRADE assessment

Table 4 presents the quality of evidence by outcome, assessed with the GRADE system. Due to serious limitations in risk of bias and publication bias, and very serious limitations in inconsistency, evidence quality was classified as moderate for TG. Also, the quality of evidence for LDL and HDL was moderate. Because of serious limitations in both inconsistency and risk of bias, the quality of the evidence was low for TC.

**Table 4:** GRADE profile of acarbose for lipid profiles

Outcomes	Risk of bias	Inconsistency	Indirectness	Imprecision	Publication Bias	WMD (95%CI)	Quality of evidence
TG	Serious limitation	Very serious limitation^a^	No serious limitation	No serious limitation	Serious limitation	−13.43 (− 19.20, −7.67)	⊕ ⊕ ⊕◯ Moderate
TC	Serious limitation	Serious limitation^a^	No serious limitation	No serious limitation	No serious limitation	−1.93 (−3.71, −0.15)	⊕ ⊕ ◯◯ Low
LDL	Serious limitation	Very serious limitation^a^	No serious limitation	Serious limitation^b^	No serious limitation	0.41 (−1.30, 2.14)	⊕ ⊕ ⊕◯ Moderate
HDL	Serious limitation	Very serious limitation^a^	No serious limitation	Serious limitation^b^	No serious limitation	0.10 (−0.69, 0.91)	⊕ ⊕ ⊕◯ Moderate

*Abbreviations*: *HDL* high density lipoprotein, *LDL* low-density lipoprotein, *TC* total cholesterol, *TG* triglyceride

^a^There is significant heterogeneity for TG (I^2^ = 86.8%), TC (I^2^ = 67.0%), LDL (I^2^ = 79.3%) and HDL (I^2^ = 87.2%)

^b^There is no evidence of significant effects of acarbose consumption on LDL and HDL

### Publication bias

**Fig. 3:**
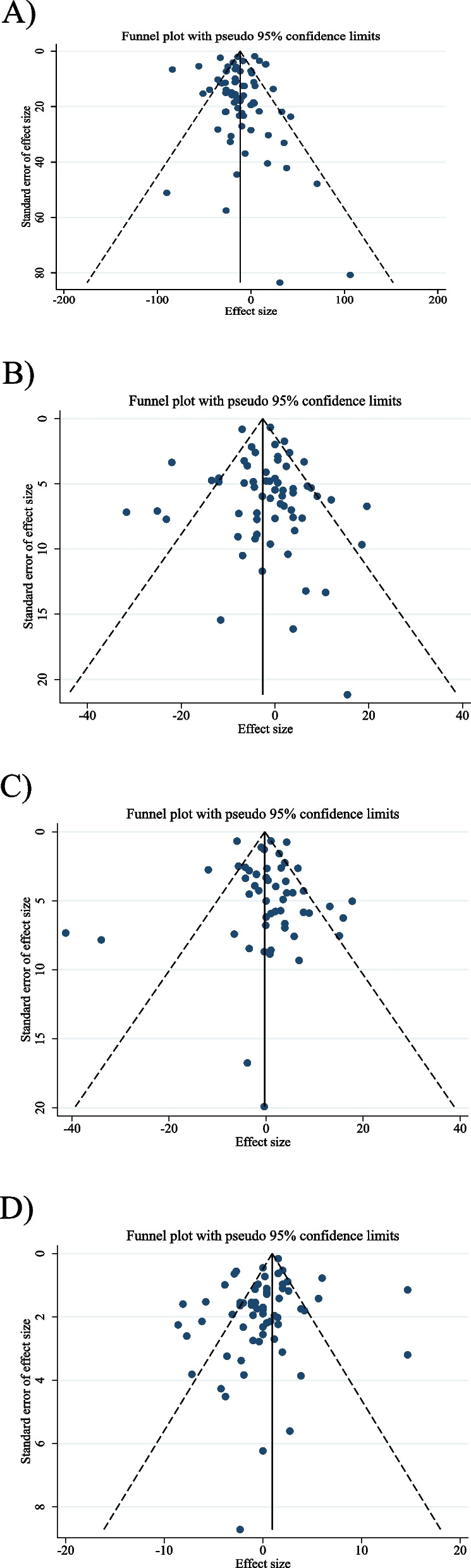
Funnel plots for the effect of acarbose on **A**) TG (mg/dl); **B**) TC (mg/dl); **C**) LDL (mg/dl) and **D**) HDL (mg/dl). TG, triglyceride; TC, total cholesterol; LDL, low-density lipoprotein; HDL, high density lipoprotein.; CI, confidence interval

The funnel plot and statistical test showed no evidence of a publication bias for TC (P _Begg’s test_ = 0.835, P _Egger’s test_ = 0.387; Fig. 3B**)** LDL (P _Begg’s test_ = 1.00, P _Egger’s test_ = 0.532; Fig. 3C**)**, and HDL (P _Begg’s test_ = 0.737, P _Egger’s test_ = 0.086; Fig. 3D). However, Begg’s test showed significant asymmetry for TG (P _Begg’s test_ = 0.019, P _Egger’s test_ = 0.630; Fig. 3A).

**Fig. 4:**
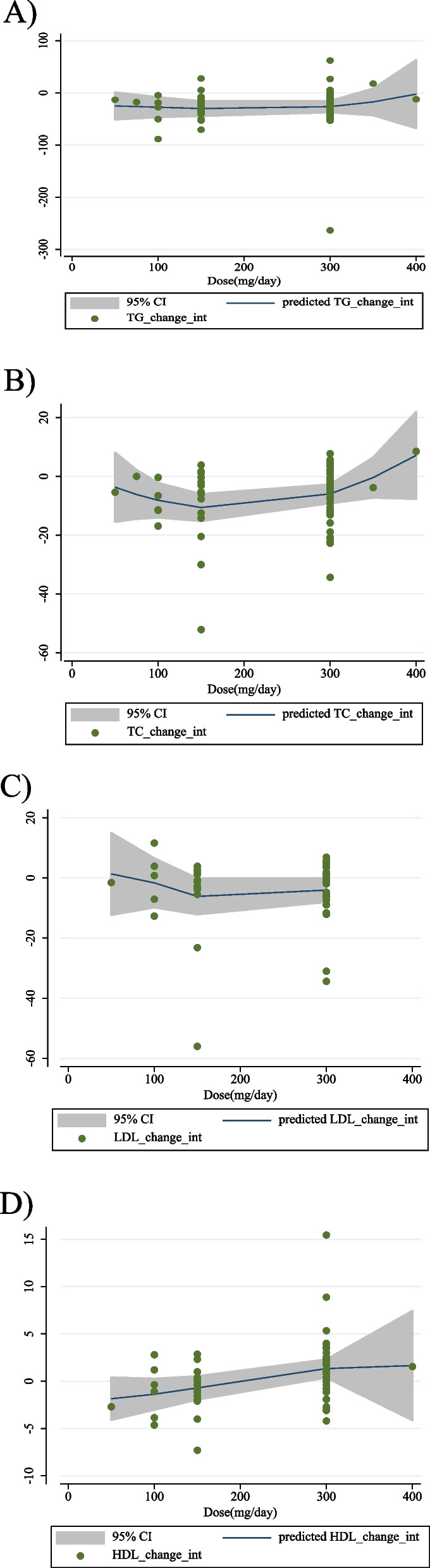
Non-linear dose-response relations between acarbose and absolute mean differences. Dose-response relations between dose (mg/d) and absolute mean differences in **A**) TG (mg/dl); **B**) TC (mg/dl); **C**) LDL (mg/dl) and **D**) HDL (mg/dl). TG, triglyceride; TC, total cholesterol; LDL, low-density lipoprotein; HDL, high density lipoprotein; CI, confidence interval

**Fig. 5:**
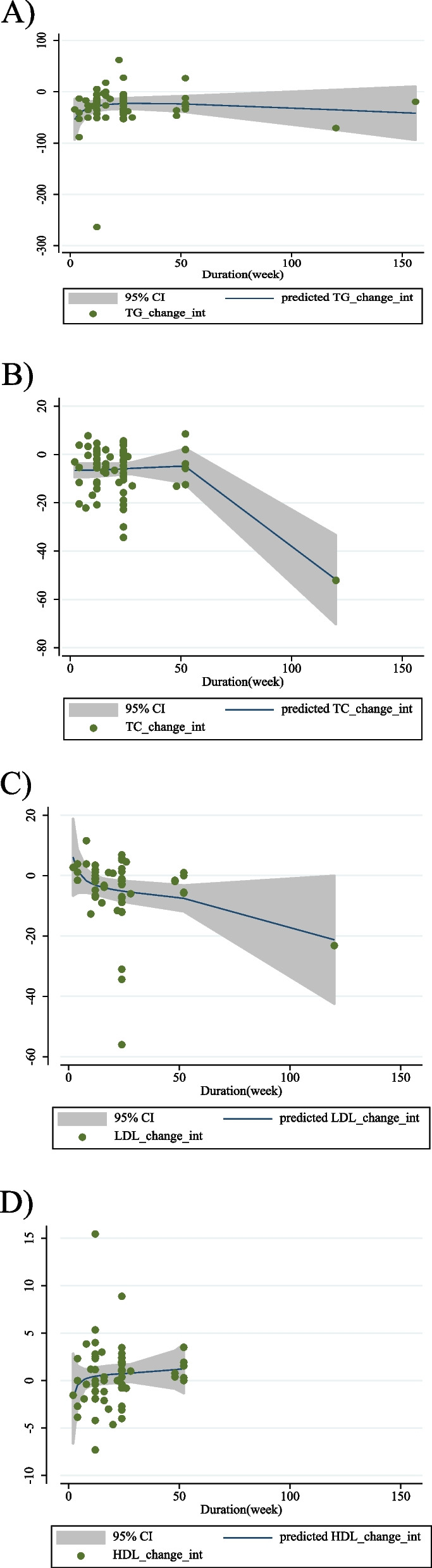
Non-linear dose-response relations between acarbose and absolute mean differences. Dose-response relations between duration of intervention (week) and absolute mean differences in **A**) TG (mg/dl); **B**) TC (mg/dl); **C**) LDL (mg/dl) and **D**) HDL (mg/dl). TG, triglyceride; TC, total cholesterol; LDL, low-density lipoprotein; HDL, high density lipoprotein; CI, confidence interval

**Fig. 6:**
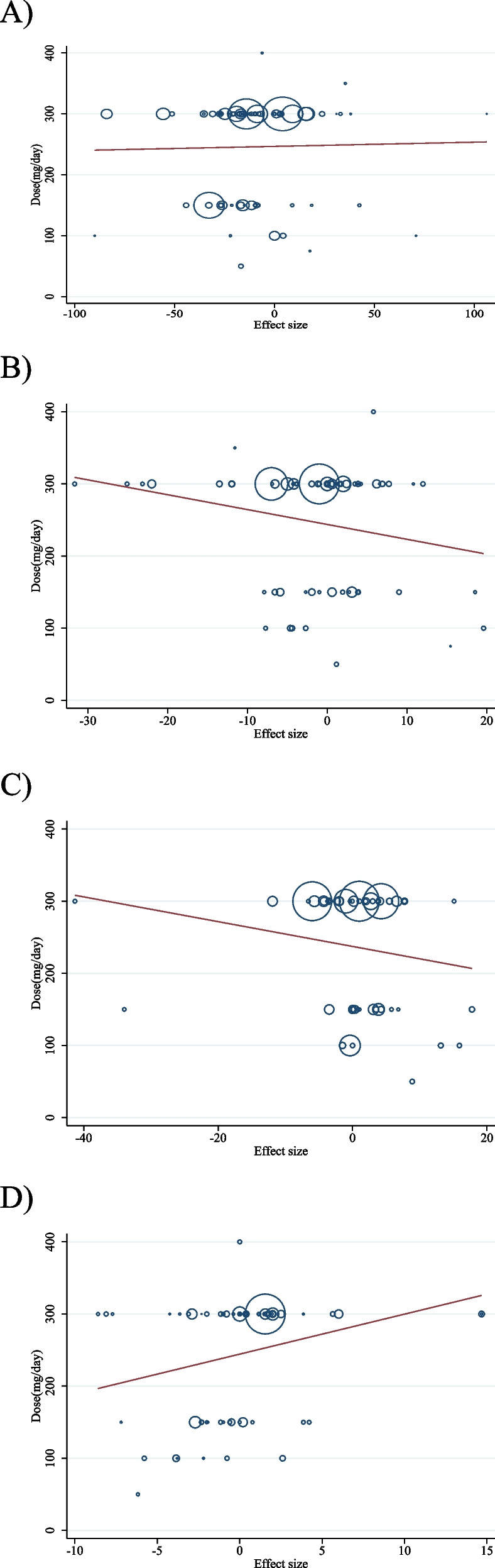
Random-effects meta-regression plots of the association between dose of acarbose (mg/d) and weighted mean difference of **A**) TG (mg/dl); **B**) TC (mg/dl); **C**) LDL (mg/dl) and **D**) HDL (mg/dl). TG, triglyceride; TC, total cholesterol; LDL, low-density lipoprotein; HDL, high density lipoprotein

**Fig. 7:**
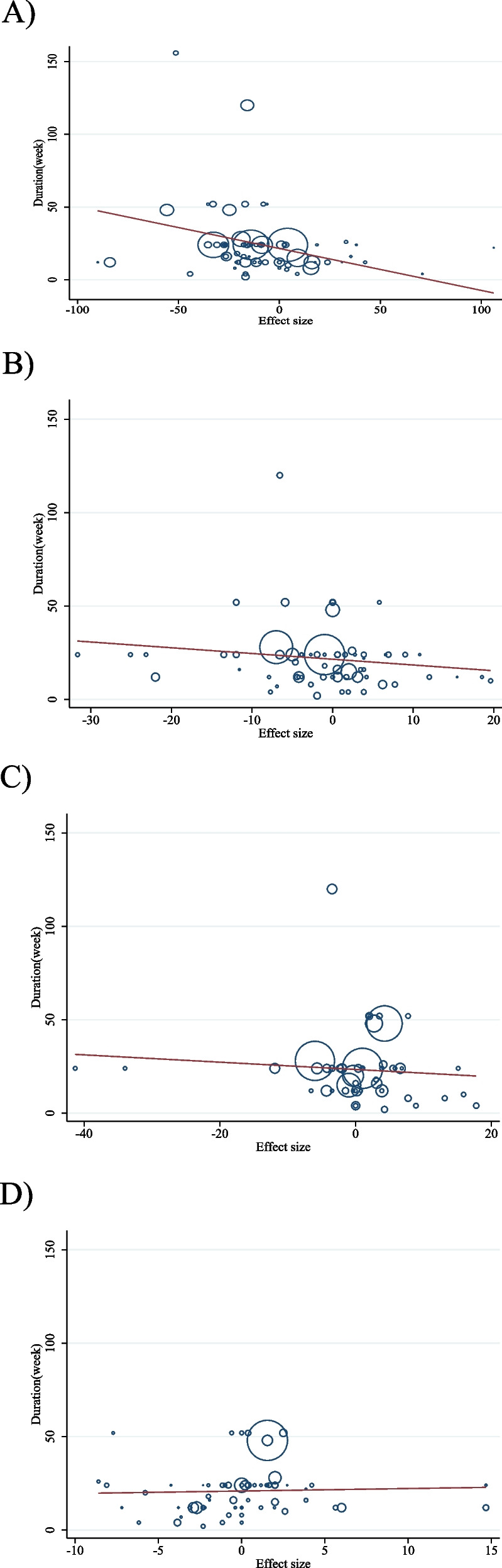
Random-effects meta-regression plots of the association between duration of intervention and weighted mean difference of **A**) TG (mg/dl); **B**) TC (mg/dl); **C**) LDL (mg/dl) and **D**) HDL (mg/dl). TG, triglyceride; TC, total cholesterol; LDL, low-density lipoprotein; HDL, high density lipoprotein; CI, confidence interval

## Discussion

This systematic review and meta-analysis provide evidence that acarbose has a significant impact on reducing TG and TC levels, while it has no significant effect on LDL or HDL. Interestingly, the beneficial effects of acarbose on TG were observed in all subgroups regardless of baseline TG levels, trial duration, intervention dose, or BMI categories, including overweight and obese individuals. Moreover, these effects were observed in both diabetic and non-diabetic patients. In terms of TC reduction, high-dose interventions (≥300 mg/d), interventions lasting ≥24 weeks, and patients with baseline TC < 200 mg/dl showed a significant reduction. Subgroup analysis also showed that low-dose interventions (< 300 mg/d) had a significant impact on lowering HDL levels. However, no relationship was found between subgroups and LDL changes. Additionally, the non-linear dose-response analysis indicated that a dosage of 400 mg/d of acarbose had a significant impact on HDL levels increment, while a duration of > 50 weeks of acarbose significantly reduced TC levels.

The findings of the present meta-analysis suggest that acarbose has a lowering effect on TG levels. This is consistent with the results of a systematic review conducted by Eleftheriadou et al., which explored the effects of various medications used for diabetes management on postprandial lipid metabolism. Their review demonstrated that acarbose can attenuate the levels of postprandial TG, chylomicrons, and very low-density lipoprotein (VLDL). It is worth noting that chylomicrons and VLDL are the primary carriers of TG in the body [100]. In a meta-analysis conducted by Va De Laar et al., acarbose was shown to lower TG levels; however, the significance of its effect was lost in the sensitivity analysis [101]. Monami et al. conducted a meta-analysis of placebo-controlled trials to assess the effects of glucose-lowering drugs on lipid profiles. They showed that acarbose could significantly reduce TG levels [15]. In line with previous studies, a systematic review by Derosa et al. reported that acarbose improved lipid profile by reducing serum TG levels [102]. However, in two systematic reviews conducted by Va De Laar et al. in 2005 and 2006, no clinically relevant effects were found on lipid profiles [103, 104].

The sub-group analysis indicated that acarbose effectively decreases TG levels, regardless of the baseline TG, intervention dose, baseline BMI, and health status (diabetic or non-diabetic). The only sub-group that did not show a reduction in serum TG levels was the one with a trial duration shorter than 24 weeks. It is possible that a trial duration shorter than 24 weeks is insufficient for acarbose to exert its effects on TG. However, additional studies are needed to confirm this finding.

Acarbose is a medication commonly used to manage T2DM and belongs to the class of α-glucosidase inhibitors (AGIs). It is a complex pseudo carbohydrate that acts as a competitor for the α-glucosidase enzymes located in the brush border of the gut epithelium. The α-glucosidase enzyme hydrolyzes complex carbohydrates to oligosaccharides in the small intestine. By competing with consumed carbohydrates, acarbose reduces α-glucosidase enzyme activity, resulting in decreased absorption of oligosaccharides and monosaccharides, which are the absorbable forms of carbohydrates. This mechanism allows acarbose to lower blood glucose levels. Although acarbose may also impact lipid profiles, data on its effects are inconclusive and require further investigation [10].

Acarbose may exert its effect on TG level by a direct action, (i.e., affecting TG synthesis in the intestine or liver, or by an indirect action) by affecting glucose and insulin level.

Carrascosa et al. conducted a study on obese diabetic Wistar rats to investigate the effects of acarbose on glucose and lipid metabolism. The results of their study showed that acarbose treatment significantly reduced TG levels when compared to untreated animals. The researchers proposed a hypothesis that acarbose’s delayed intestinal uptake of carbohydrates could lead to a reduced availability of substrates required for TG synthesis, ultimately resulting in a decreased rate of TG synthesis [105]. Studies have indicated that acarbose treatment leads to a decrease in chylomicron remnant production by impairing TG synthesis in the small intestine [44]. Another study by Krause et al. found that the reduction in TG levels by acarbose is due to a decrease in VLDL synthesis and secretion, with no effects on TG removal from the bloodstream [106]. Acarbose may also influence apolipoprotein levels, which can affect the activity of lipoprotein lipase (LPL). Modulation of LPL activity may also contribute to the TG-lowering effect of acarbose [107].

Elevated serum glucose and insulin concentrations are known to promote hypertriglyceridemia [108, 109]. As acarbose lowers glucose levels, it may indirectly affect TG levels. Acarbose improves insulin resistance, which in turn reduces free fatty acid (FFA) levels by inhibiting peripheral lipolysis, as insulin has antilipolytic effects. Reduced FFA synthesis leads to a decrease in VLDL synthesis in the liver. Given that VLDL is the primary carrier of TG, this can lead to a reduction in TG levels [110].

It has also been proposed that acarbose may impact serum lipid profile through its effects on body weight. In a meta-analysis by Li et al., it was demonstrated that acarbose monotherapy resulted in weight loss compared to the control group [111]. As weight has a significant influence on serum lipid profile, with obesity increasing TG and TC levels and decreasing HDL levels, acarbose may indirectly lower TG levels through its ability to induce weight loss [112, 113].

The results of this meta-analysis regarding HDL were inconclusive, and acarbose had no significant effect on HDL in pooling effect size. In a meta-analysis conducted by Van de laar et al. in 2005, acarbose intake did not affect HDL levels [101]. In two other systematic reviews conducted by Van de laar et al. in 2005 and 2006, acarbose had no clinically relevant effects on lipid profile [103, 104]. However, a meta-analysis of placebo-controlled trials conducted by Monami et al. showed that HDL levels could be increased by acarbose intake [15].

It was found in our meta-analysis that acarbose was effective in lowering HDL levels only when the intervention dose was lower than 300 mg/d, as revealed by subgroup analysis. However, the underlying mechanism by which acarbose decreases serum HDL levels remain unclear and requires further investigation.

The non-linear dose-response analysis revealed an optimum effect of acarbose dose (400 mg/d) on serum HDL level.

This meta-analysis revealed that acarbose intake reduces serum TC levels. In a meta-analysis conducted by Van de laar et al. in 2005, acarbose did not affect TC levels [101]. In addition, another meta-analysis of placebo-controlled trials was not conclusive about the effect of acarbose on TC levels [15]. In two systematic reviews conducted by Van de laar et al. in 2005 and 2006, no clinically relevant effects were found on lipid profiles [103, 104]. However, in a systematic review conducted by Derosa et al., acarbose was shown to be effective in lowering TC levels [102].

Subgroup analysis revealed that acarbose was more effective in reducing serum TC levels when either its intervention dose was higher than 300 mg/d or its trial duration was longer than 24 weeks. In addition, it was more effective in reducing serum TC levels when participants had diabetes, were overweight, or in cases where their baseline TC was lower than 200 mg/dl. Being more effective in higher dosages and longer interventions could be explained by acarbose bioavailability in the small intestine of participants. On the other hand, acarbose was more effective in lowering TG in obese and diabetic patients. These patients have higher glucose levels and probably have higher levels of insulin resistance. Acarbose could lower TC levels by lowering blood glucose and improving insulin sensitivity in these patients.

The non-linear duration-response analysis revealed a significant association between acarbose intake duration and TC levels. Although TC levels were not changed when the duration of intervention was shorter than 50 weeks, a longer duration of intervention drastically reduced TC levels.

One of the mechanisms proposed for the cholesterol-lowering effect of acarbose is its effect on enhancing bile excretion in the small intestine. However, further investigations are needed to confirm this mechanism [114]. Since acarbose delays carbohydrate digestion and absorption, it also affects short-chain fatty acid absorption and increases fecal excretion [115]. Acarbose exerts its effect by delaying the hydrolysis of carbohydrates and increasing the flow of these carbohydrates to the large intestine. This overflow of undigested carbohydrates changes the structure and function of gut microbiota and increases the fecal production of short-chain fatty acids (SCFA) [116, 117]. Acetate, propionate, and butyrate are the three important SCFAs. Inulin is a polysaccharide that is used as a prebiotic. Inulin injection has been shown to reduce TG, TC, and LDL levels by increasing acetate, propionate, and butyrate production in the gut. SCFAs promote fatty acid oxidation and inhibit fatty acid synthesis in the liver and lipolysis in adipose tissue. These effects of SCFAs could be mediated by activating the adenosine monophosphate-activated protein kinase (AMPK), a central regulator in energy homeostasis [118]. Also, it is known that propionate inhibits the utilization of acetate for lipid and cholesterol synthesis. Therefore, acarbose can lower the cholesterol level by increasing SCFAs production in the large intestine [119].

As explained in the previous paragraph, studies have demonstrated that acarbose can decrease VLDL production in the liver. Given the established link between changes in TG and cholesterol levels, it is plausible to hypothesize that the reduction in VLDL production may be the underlying mechanism by which acarbose lowers cholesterol levels [115]. Another possible mechanism by which acarbose could lower cholesterol levels is through the normalization of the activity of hepatic 3-hydroxy-3-methylglutaryl Co-A (HMG Co-A) synthase. This enzyme plays a crucial role in cholesterol synthesis [120]. Acarbose could also affect cholesterol levels through indirect mechanisms. As mentioned in previous sections, acarbose has a lowering effect on body mass [111]. Since obesity is associated with serum lipid profile, weight loss induced by acarbose could reduce cholesterol levels [112, 113]. Together, these mechanisms can explain the lowering effects of acarbose on TG and TC. However, the effect of acarbose on HDL and LDL and the mechanisms by which acarbose affects these lipoproteins are not entirely understood. Therefore, further investigations need to be carried out to elucidate these matters.

This systematic review and meta-analysis have identified several important limitations that require attention. Chief among these is the quality of the included studies, as our risk of bias assessment found many studies to have a high risk of bias, resulting in low or moderate quality of evidence. While most of the studies were randomized double-blind with control groups, the risk of bias may still affect the validity of the meta-analysis. Therefore, more well-designed studies are required to establish the true effects of acarbose on lipid profile. Moreover, lipid profile was a secondary finding in most studies, with the primary focus on glucose metabolism, potentially leading to underreporting of data and bias. When interpreting the results, it is crucial to consider the heterogeneity in participants’ age, BMI, and health status, although we attempted to address this through subgroup analysis. Furthermore, variations in laboratory methods and biochemical assay kits for lipid profile measurement may introduce intra- and inter-assay variation and bias the results’ interpretation. Another limitation of this study is that the control group was not the same and there were different drugs compared to acarbose, which could affect the results. Hence, more large-scale, rigorously controlled clinical trials are needed to further elucidate the effects of acarbose on lipid profile. Despite these limitations, several strengths of this study should be acknowledged. Firstly, this is the first systematic review and meta-analysis to focus specifically on the effects of acarbose on lipid profile, providing a comprehensive view of the impact of acarbose on TG, TC, HDL, and LDL. Secondly, the review did not limit the publication date or language, making it a comprehensive study. Additionally, the included studies spanned different regions globally, enhancing the generalizability of the results to adult populations with both healthy and unhealthy statuses. The standardized methodology and various statistical methods employed in this study provided a robust assessment of the effect of acarbose on serum lipid profile, and further sub-group analyses, GRADE and sensitivity assessments, and dose and duration-response analyses were conducted to determine the actual impact of acarbose on lipid profile. The study also collected all adverse effects mentioned in trials. Overall, while this systematic review and meta-analysis offer a comprehensive view of the effects of acarbose on lipid profile, more large-scale, rigorously controlled clinical trials with a primary focus on the effects of acarbose on lipid profile are needed to establish conclusive evidence.

## Conclusion

This meta-analysis provides evidence that acarbose is effective in lowering TG and TC levels, but its effects on LDL and HDL are inconclusive. The dose-response analysis indicates that HDL levels increase gradually with increasing doses of acarbose ranging from 50 to 400 mg/d. Moreover, the duration-response analysis reveals that longer intervention periods substantially reduce serum TC levels. Acarbose may exert its lipid-lowering effects through a direct mechanism by modulating lipid synthesis and secretion or an indirect mechanism by reducing blood glucose levels and improving insulin sensitivity. However, further well-designed randomized controlled trials and mechanistic studies are needed to elucidate the effects of acarbose on HDL and LDL.

## Data Availability

The datasets analyzed during the current study are presented in the manuscript.

## Abbreviations

RCTs: Randomized controlled trials
SEM: Standard Error of Mean
TC: Total cholesterol
TG: Triglyceride
LDL: Low-density lipoprotein
HDL: High-density lipoprotein
PCSK9: Proprotein convertase subtilisin/kexin type 9
PICO: Participant, Intervention, Comparison/Control, Outcome
GRADE: Grading of Recommendations Assessment, Development, and Evaluation
WMD: Weighted mean difference
HMG-CoA: 3-hydroxy-3-methylglutaryl-CoA
BMI: Body mass index
WC: Waist circumference
SDs: Standard deviations
SEM: Standard Error of Mean
I^2^: I square
PRISMA: Preferred Reporting Items for Systematic Reviews and Meta-Analysis
SE: Standard error
SD: Standard deviation
VLDL: Very low-density lipoprotein
AGIs: α-glucosidase inhibitors
FFA: Free fatty acid
LPL: Lipoprotein lipase activity
SCFA: Short-chain fatty acids
AMPK: Adenosine monophosphate-activated protein kinase
